# Biostatistical challenges in multicenter clinical trials: Best practices for collaboration and data harmonization across institutions

**DOI:** 10.6026/973206300212935

**Published:** 2025-08-31

**Authors:** Deepankar Satapathy, Madhavi Eerike, Sanket Mishra, Sandip Dhole, U. Rekha Priyadarshini, Bhagabati Prasad Dash, Debasis Bisoi

**Affiliations:** 1 Department of Orthopaedics, AIIMS, Bibinagar, Hyderabad, India; 2 Department of Pharmacology, AIIMS, Bibinagar, Hyderabad, India; 3 Department of Orthopaedics, IMS and SUM Hospital, Bhubaneswar, India; 4 Department of Physical Medicine and Rehabilitation, AIIMS, Bibinagar, Hyderabad, India; 5 Department of Orthodontics, Kalinga Institute of Dental Sciences, KIIT Deemed to be University, Bhubaneswar, India

**Keywords:** Artificial Intelligence, Cloud-Based platforms, CONSORT guidelines, data standardization, emerging technologies, mobile health applications, multicentre orthopaedic trials

## Abstract

Multicentre orthopaedic clinical trials present distinct biostatistical and operational challenges that can affect the reliability and generalizability of their findings. Differences in site protocols, patient demographics, outcome measurements and regulatory environments contribute to data variability and complexity. This review discusses key strategies to address these issues, including the implementation of multilevel statistical models, development of uniform data collection tools and centralized processes for outcome assessment. Emphasis is placed on the value of early, coordinated planning and sustained collaboration among participating centers to ensure consistency and quality across sites. The review also explores the potential of digital technologies to streamline data integration and support harmonized workflows. By promoting methodological rigor and cross-institutional cooperation, these approaches aim to enhance the validity and impact of orthopaedic multicentre trial outcomes.

## Background:

Clinical trials are systematic investigations designed to evaluate the safety and efficacy of medical, surgical, or rehabilitative interventions in human participants [1]. In orthopaedics, clinical trials are crucial for advancing surgical techniques, implant technologies, rehabilitation protocols and pain management strategies [2]. Multicenter clinical trials conducted across multiple institutions using a unified protocol have become increasingly important in orthopaedic research to ensure robust, generalizable and clinically relevant findings [3]. These trials enhance patient recruitment, promote geographic and demographic diversity and allow for more rapid evidence generation [1]. In orthopaedics conducting these are critical due to variability in surgical technique, postoperative care and patient anatomy which can significantly affect treatment outcomes [4]. Multicenter trials commonly involve large sample sizes, centralized coordination, stratified randomization and standardized outcome measures [5]. They are essential for studying interventions in orthopaedics where rare outcomes, such as implant failure or surgical complications, require broad patient populations for adequate statistical power [6]. Additionally, the inclusion of both academic and community hospitals reflects real-world clinical heterogeneity, making findings more applicable across healthcare systems [7]. Despite these advantages, multicenter orthopaedic trials pose unique biostatistical and operational challenges. Differences in surgeon expertise, inconsistent radiographic assessments, variable endpoint definitions (e.g., union vs. malunion) and protocol deviations across sites can compromise data integrity and comparability [8]. Heterogeneous data collection systems, missing data and fragmented reporting further complicate statistical analysis and necessitate robust harmonization and coordination strategies [9]. The bio statistical and operational challenges in interventional studies have been reviewed by various authors. However since the challenges in multicentric orthopaedic trials are unique in their own way they have not been discussed in the previous reviews. In this context this review explores the key biostatistical challenges encountered in multicenter orthopaedic clinical trials and presents best practices to address them. Therefore, it is of interest to describe the key biostatistical challenges in multicenter orthopaedic clinical trials and outline effective strategies for overcoming them, ensuring high-quality, reliable, and generalizable outcomes across diverse settings.

## Review:

## Biostatistical challenges in multicenter orthopaedic clinical trials:

Multicenter orthopaedic trials introduce several layers of statistical complexity due to the involvement of diverse clinical sites, heterogeneous patient populations and varied surgical practices. This section outlines the key biostatistical challenges that researchers must address to ensure valid, and interpretable results [10] reliable.

## Site heterogeneity and inter-surgeon variability:

One of the most significant challenges in multicenter trials is managing variability between sites. Differences in surgeon experience, institutional protocols, patient demographics and regional practices can create non-random variation that affects outcomes [3]. Failure to account for such heterogeneity may lead to biased estimates or inflated Type I error rates. Statistical techniques such as mixed-effects models and hierarchical (multilevel) modelling can help address between-site variability by appropriately nesting data and adjusting for cluster-level effects. These models allow researchers to examine fixed effects (treatment) and random effects (site or surgeon), providing more accurate estimates of intervention efficacy [11].

## Sample size and power considerations:

Designing a well-powered multicenter orthopaedic trial requires careful consideration of intra-class correlation (ICC), which reflects the similarity of responses within the same center [8]. Ignoring clustering can lead to underpowered studies and misleading conclusions. Adjusting for ICC in sample size calculations often through design effects is critical. Additionally, stratified randomization by site may help reduce imbalances in prognostic factors and improve statistical efficiency [9].

## Inconsistent outcome measurement and endpoint definition:

Orthopaedic outcomes often rely on a combination of subjective and objective measures, including radiographic interpretation, patient-reported outcomes and physical function assessments. Variability in the interpretation of these measures across sites can threaten both reliability and validity [10]. Establishing centralized adjudication committees or using core laboratories for radiographic outcomes can improve consistency. Implementing validated scoring systems for patient related outcome measures (e.g., DASH, SF-36 and WOMAC) across all sites enhances comparability [12].

## Missing data and protocol deviations:

Missing data are a common issue in orthopaedic trials, especially when follow-up is prolonged or when assessments require in-person visits. Protocol deviations, such as off-label use of implants or variations in rehabilitation protocols, further complicate the analytic framework [13]. Appropriate imputation methods, such as multiple imputation or sensitivity analyses using pattern-mixture models; can mitigate bias introduced by missing data. Predefining protocol adherence thresholds and monitoring deviations closely are crucial for maintaining data quality and trial integrity [13, 14].

## Multiplicity and subgroup analyses:

Multicenter trials often involve multiple outcomes and subgroup analyses (e.g., age, fracture type, comorbidities), increasing the risk of Type I errors due to multiple comparisons [15, 16]. A systematic review analysed articles from two major orthopaedic journals and found that numerous statistical tests were frequently performed without appropriate corrections, leading to an increased risk of Type I errors [17]. The authors emphasized the importance of correcting for multiple comparisons to reduce the likelihood of false-positive findings in multicenter trials. Adjustments using Bonferroni correction, false discovery rate control, or pre-specification of primary and secondary endpoints help control for multiplicity. Clear distinction between confirmatory and exploratory analyses should be maintained to avoid over interpretation of findings [18]. There are limitations of the Bonferroni correction in controlling the false positive rate when performing multiple hypothesis tests in health and medical studies. It is important to distinguish between exploratory and hypothesis-driven analyses to avoid over interpretation of findings. The use of false discovery rate (FDR) control is a more powerful and appropriate method for addressing multiplicity in such studies [19]. Table 1 (see PDF) summarises the biostatistical challenges and best practices in multicenter orthopaedic clinical trials, covering key areas such as site heterogeneity, sample size estimation, outcome measures, and more. Figure 1 (see PDF) illustrates the various biostatistical challenges faced in multicenter orthopaedic clinical trials, offering a visual representation of the complexity involved.

## Best practices for statistical collaboration in multicenter orthopaedic trials:

Effective statistical collaboration is key to the success of multicenter orthopaedic clinical trials. This can be achieved by early involvement of biostatisticians and maintaining continuous communication between research teams across institutions to ensure methodological rigor, efficient trial conduct and reproducible outcomes. This section outlines best practices for fostering productive statistical collaboration in multicenter settings [20]. Best practices for statistical collaboration in multicenter orthopaedic trials are shown in Figure 2 (see PDF).

## Early engagement of biostatisticians:

Biostatisticians should be involved from the earliest phases of trial planning, including protocol development, outcome selection, and sample size estimation and randomization strategy. Early input allows for better anticipation of site-level variability, missing data risks and analysis requirements. Collaborative protocol writing also ensures that statistical methods align with clinical objectives and regulatory expectations, minimizing the need for protocol amendments later in the trial [21].

## Development of a statistical analysis plan (SAP):

A well-defined, pre-specified SAP should outline all primary and secondary endpoints, statistical models, handling of missing data, adjustments for multiplicity and prespecified subgroup analyses. In multicenter trials, the SAP must also address hierarchical data structures, inter-site variability and stratification strategies. Finalizing the SAP before data unblinding reduces bias and supports transparency and credibility in reporting [22].

## Use of coordinating centers and central statistical teams:

Establishing a central coordinating center with a dedicated statistical team ensures consistency in data handling, quality checks and interim analyses across all participating sites [21, 22- 23]. The statistical coordinating center can serve as the main hub for:

[1] Generating randomization schedules

[2] Monitoring recruitment and protocol adherence

[3] Managing database locking procedures

[4] Conducting blinded data reviews and interim analyses

Such centralized oversight also facilitates rapid troubleshooting and unified reporting [23].

## Regular communication and training across sites:

Regular virtual or in-person meetings involving clinicians, statisticians and data managers across sites promote real-time problem solving, consistent protocol implementation and unified decision-making [24, 25- 26]. Statistical training sessions can help site investigators understand the trials analytic approach, which enhances protocol compliance and interpretation of results during dissemination.

## Transparent reporting and collaborative publication:

Multicenter trials benefit from collaborative writing groups, which include biostatisticians and investigators from multiple sites. Following CONSORT guidelines for multicenter studies and including detailed statistical methods sections supports reproducibility and transparency [27, 28-29]. Clear reporting of statistical assumptions, missing data handling and site-specific effects enables readers to evaluate the trial's validity and applicability to their clinical context.

## Emerging technologies in multicenter orthopaedic trials:

The integration of emerging technologies into multicenter orthopaedic clinical trials has the potential to streamline workflows, enhance data quality, improve patient engagement and increase the scalability of research efforts. From digital health platforms to artificial intelligence (AI), these innovations are reshaping how trials are designed, conducted and analyzed [30].

## Electronic health record (EHR) integration:

Linking trial databases with EHR systems allows for automated extraction of clinical data, reducing transcription errors and workload at research sites [1]. EHR integration enables real-time monitoring of patient eligibility, adverse events and longitudinal outcomes, which is particularly valuable in long-term orthopaedic studies like implant survival or reoperation rates [2]. Standardized health data models such as OMOP and HL7 FHIR are increasingly used to harmonize EHR-derived data across institutions [30, 31- 32]. Observational Medical Outcomes Partnership (OMOP) is a public-private partnership that developed the Common Data Model (CDM), a standardized format for storing and analyzing observational health data. The OMOP CDM helps facilitate research across different healthcare organizations by providing a shared language for data. HL7 FHIR stands for Health Level 7 Fast Healthcare Interoperability Resources. It is a next-generation standard for exchanging healthcare information electronically, developed by HL7 International. FHIR is designed to enable the quick and efficient exchange of health data, including clinical and administrative data.

## Electronic consent (eConsent) and remote enrollment:

E-Consent platforms allow patients to review study information, ask questions and provide informed consent remotely. These tools can improve comprehension through multimedia elements and facilitate recruitment from geographically diverse populations particularly beneficial in orthopaedic trials where patient travel may be limited post-surgery. Remote enrollment also enhances trial accessibility and helps reduce recruitment timelines in large, multicenter studies [33, 34 35-36].

## Wearable devices and mobile health (mHealth) apps:

Wearable technologies (e.g., smart watches, gait trackers) and mHealth apps provide continuous, real-world data on functional recovery, mobility and physical activity [37]. A scoping review published in the Journal of Orthopaedic Surgery and Research examined the use of wearable devices for postoperative monitoring in hip, knee and spine surgeries. The study highlighted that devices like Fitbit, Xiaomi Mi Band and Acti Graph are employed to monitor gait and mobility metrics, providing continuous, real-world data on functional recovery. This approach reduces reliance on traditional clinical visits and enhances data richness [38]. This is particularly relevant in orthopaedic trials assessing joint replacement outcomes or fracture rehabilitation. These tools enable objective monitoring outside clinical settings and can reduce reliance on site visits or self-reported measures, improving adherence and data richness.

## AI and machine learning for image and data analysis:

Artificial Intelligence (AI) tools are being used for automated interpretation of radiographic outcomes, including fracture healing, implant positioning and alignment. These algorithms reduce inter-observer variability and support centralized adjudication with high consistency. Machine learning models can also assist in predictive analytics, patient risk stratification and adaptive trial design, improving trial efficiency and personalization [39, 40].

## Cloud-Based platforms and real-time dashboards:

Cloud-based data management platforms enable secure, centralized storage and real-time access to data across institutions [41]. Dashboards allow investigators and sponsors to track recruitment, data completeness, protocol deviations and adverse events in real time. For instance, Cloudbyz offers a platform that includes customizable dashboards to monitor metrics such as patient recruitment, data completeness, site performance and safety signals. These dashboards enable stakeholders to track trial progress and identify trends or bottlenecks in real time. Additionally, the platform provides advanced analytics and reporting tools to analyze patient demographics, efficacy trends, adverse events and other critical data, facilitating informed decision-making. Similarly, LabKey provides a centralized platform designed to harmonize participant, sample and results data. It offers secure access, regulatory compliance and efficient data integration, ensuring data quality and accessibility at every stage of trial. These systems promote transparency; facilitate communication among sites and support rapid decision-making during interim analyses [42]. Figure 3 (see PDF) shows schematic diagram of a multicenter trial organizational framework.

## Discussion:

Multicenter orthopaedic clinical trials are indispensable for generating robust, generalizable evidence to guide surgical practice, implant innovation and rehabilitation strategies. While these trials offer numerous advantages such as enhanced patient diversity, faster recruitment and broader applicability they also pose substantial biostatistical and operational challenges. Variability across sites, complex data structures, missing data and inconsistent outcome definitions demand sophisticated statistical methods, proactive collaboration and rigorous data harmonization [43]. The integration of centralized coordination centers, standardized data collection protocols and validated measurement tools has improved the reliability of multicenter trials. Moreover, early and sustained involvement of biostatisticians ensures methodological rigor throughout all trial phases, from design to dissemination. Technological advancements including EHR integration, remote monitoring, wearable devices and artificial intelligence are further transforming the landscape, enabling more efficient, patient-centered and scalable trials [45]. Looking ahead, several opportunities exist to enhance the design and conduct of multicenter orthopaedic research. Adaptive and platform trial designs may provide more flexible, efficient evaluation of multiple interventions within a single framework. Increasing adoption of real-world data, decentralized trial models and data-sharing consortia will also help accelerate innovation and support regulatory decision-making [44].

Artificial intelligence (AI) and the Metaverse and Augmented Reality hold immense potential in transforming the future of multicenter orthopaedic clinical trials. AI can streamline data analysis, enabling the integration of vast amounts of patient data from diverse clinical settings, while also addressing challenges such as missing data and inconsistent outcome definitions through advanced machine learning algorithms [45]. AI can automate patient recruitment processes, optimize trial designs and predict trial outcomes, which can significantly enhance efficiency and reduce the time and cost associated with trials [49]. The Metaverse, on the other hand, can provide immersive virtual environments for remote monitoring, virtual clinical visits and rehabilitation simulations, offering a more accessible and patient-centered approach. It can also facilitate real-time data sharing and collaboration across global sites, overcoming geographical barriers [46, 47]. Together, AI and the Metaverse can help overcome the biostatistical and operational hurdles faced by traditional trials, leading to faster and more reliable clinical outcomes and ultimately improving the quality of patient care and advancing research [48, 49]. Ultimately, the success of multicenter orthopaedic trials depends on interdisciplinary collaboration, transparency and a commitment to methodological excellence. By embracing best practices and emerging technologies, the orthopaedic research community can overcome existing barriers and deliver high-quality evidence that translates into improved patient outcomes across diverse care settings.

## Conclusion:

Multicenter orthopaedic trials are crucial for advancing surgical practices and patient care, despite the biostatistical and operational challenges they present. By integrating standardized protocols, centralized coordination and early involvement of biostatisticians, these trials can generate more reliable and generalizable evidence. The future of orthopaedic research lies in embracing innovative trial designs, real-world data and emerging technologies to improve trial efficiency and patient outcomes.
